# Insights into the Genetic and Epigenetic Landscape of Endocrine Autoimmunity: A Systematic Review

**DOI:** 10.3390/genes16121506

**Published:** 2025-12-16

**Authors:** Gerdi Tuli, Jessica Munarin, Katherine Stephanie Davalos Flores, Luisa De Sanctis

**Affiliations:** 1 Department of Pediatric Endocrinology, Regina Margherita Children’s Hospital, 10124 Turin, Italy; jessica.munarin@unito.it (J.M.); katherinestephanie.davalosflores@unito.it (K.S.D.F.); luisa.desanctis@unito.it (L.D.S.); 2 Department of Pediatrics, University of Turin, 10124 Turin, Italy

**Keywords:** endocrine autoimmune diseases, genetics, epigenetics, thyroid, pituitary, parathyroid, adrenal, gonadal

## Abstract

Background/Objectives: Endocrine autoimmune diseases, including autoimmune thyroid, pituitary, parathyroid, adrenal, and gonadal diseases, result from complex interactions between genetic susceptibility and environmental triggers. Advances in genomics and epigenomics have provided novel insights into the molecular pathways leading to immune dysregulation and endocrine tissue destruction. This review summarizes recent progress in understanding the genetic and epigenetic bases, emphasizing shared and disease-specific mechanisms that contribute to autoimmunity and endocrine dysfunction. Methods: A comprehensive literature search was performed in PubMed, Scopus, and Web of Science up to August 2025, focusing on genome-wide association studies (GWAS), next-generation sequencing, and epigenetic profiling (DNA methylation, histone modification, and non-coding RNA regulation). Results: More than 60 susceptibility loci have been identified across endocrine autoimmune diseases (EADs), including key genes in immune tolerance (HLA, CTLA4, PTPN22) and endocrine-specific pathways. Epigenetic studies reveal that altered DNA methylation and histone acetylation patterns in immune and endocrine cells modulate gene expression without changing the DNA sequence, linking environmental exposures to disease onset. Dysregulated microRNAs further influence immune signaling and cytokine networks. Conclusions: Genetic and epigenetic discoveries highlight the multifactorial nature of EADs and reveal potential biomarkers for early detection and targets for precision immunotherapy. Future research integrating multi-omics and longitudinal analyses will be crucial to unravel causal mechanisms and develop personalized preventive strategies.

## 1. Introduction

Autoimmune diseases occur when the immune system mistakenly attacks the body’s own tissues, leading to chronic inflammation, tissue damage, and dysfunction that may be systemic or organ specific. These conditions encompass a wide spectrum of disorders, including autoimmune thyroid disease, hypophysitis, autoimmune parathyroid disease, and Addison’s disease. Other non-endocrine disorders include the nervous, cardiovascular, respiratory, and digestive systems; skin; kidney; and blood cells.

Endocrine autoimmune diseases (EADs) encompass both common and rare entities. Autoimmune thyroid diseases (AITD), including Graves’ disease (GD) and Hashimoto’s thyroiditis (HT), are among the most frequent autoimmune disorders, affecting approximately 5% of the population [1]. In contrast, primary adrenal insufficiency affects about 100 per million individuals in Europe, while primary (autoimmune) hypophysitis is much rarer, with an estimated incidence of roughly 1 per 9 million per year [2,3]. Although their clinical presentations differ, these conditions share a common genetic susceptibility framework. Monogenic autoimmune polyendocrine syndrome type 1 (APS-1), or autoimmune polyendocrinopathy-candidiasis-ectodermal dystrophy (APECED), caused by *AIRE* mutations, exemplifies a lifelong predisposition to multiple endocrine autoimmune targets, notably hypoparathyroidism and Addison’s disease. Furthermore, common *AIRE* variants and immune-regulatory loci (e.g., *HLA*, *CTLA4*, *PTPN22*) contribute polygenically to disease risk in the general population [4,5,6,7,8,9].

Genetic studies have highlighted the contribution of susceptibility loci within immune-regulatory pathways, particularly genes encoding HLA molecules, costimulatory receptors, and transcription factors essential for immune tolerance (Table 1).

Copy-number or single-nucleotide variations in loci such as PTPN22, CTLA4, and FOXP3 and thyroid-specific antigens (TSHR, TPO, TG) are implicated across several autoimmune entities. These loci encode proteins involved in key immune tolerance mechanisms, such as T-cell activation and regulation, costimulatory signaling, and maintenance of self-tolerance by regulatory T-cells. Variants in *PTPN22* and *CTLA4*, for example, alter signaling thresholds in lymphocytes, predisposing them to aberrant immune activation, while mutations in *FOXP3* can impair regulatory T-cell function. Similarly, polymorphisms affecting *TSHR*, *TPO*, and *TG* may enhance the presentation of thyroid autoantigens, promoting thyroid-specific autoimmunity. The involvement of these shared genetic pathways helps to explain the well-recognized clustering of autoimmune disorders within individuals and families and the tendency for patients with autoimmune thyroid disease to develop additional autoimmune conditions, such as type 1 diabetes, celiac disease, or autoimmune adrenalitis [9,10,11,12,13,14,15,16]. In AITD, twin, family, and segregation data further underscore a substantial heritable component while also revealing an overlap between GD and HT within kindreds [1].

Epigenetic alterations, including DNA methylation and histone modifications, are emerging as key modulators of immune tolerance and effector function. Disease-specific methylation signatures have been described in AITD and Addison’s disease, and epigenetic programs likely mediate environmental influences such as smoking, iodine exposure, infection, and endocrine-disrupting chemicals [13,15,17,18,19]. These findings provide a conceptual bridge linking inherited susceptibility with dynamic environmental cues.

The aim of this work is to synthesize genetic and epigenetic mechanisms across major endocrine autoimmune diseases (AITD, hypophysitis, autoimmune parathyroid disease, and Addison’s disease), highlighting both shared and disease-specific pathways of immune dysregulation. Principal conclusions emphasize (i) convergent immune-regulatory genetics (HLA, checkpoints, AIRE) that create a common risk scaffold, (ii) epigenetic programs that shape organ selectivity and treatment response, and (iii) clinically actionable implications for risk prediction and targeted therapy.

## 2. Materials and Methods

We conducted a literature search on the Pubmed and Embase databases, with a timeframe from 2010 to September 2025. Only English language articles were included. All types of articles were included as long as the studies were relevant for future studies in humans.

We performed an electronic search using database-specific keywords (see Table 1).

**Figure 1:**
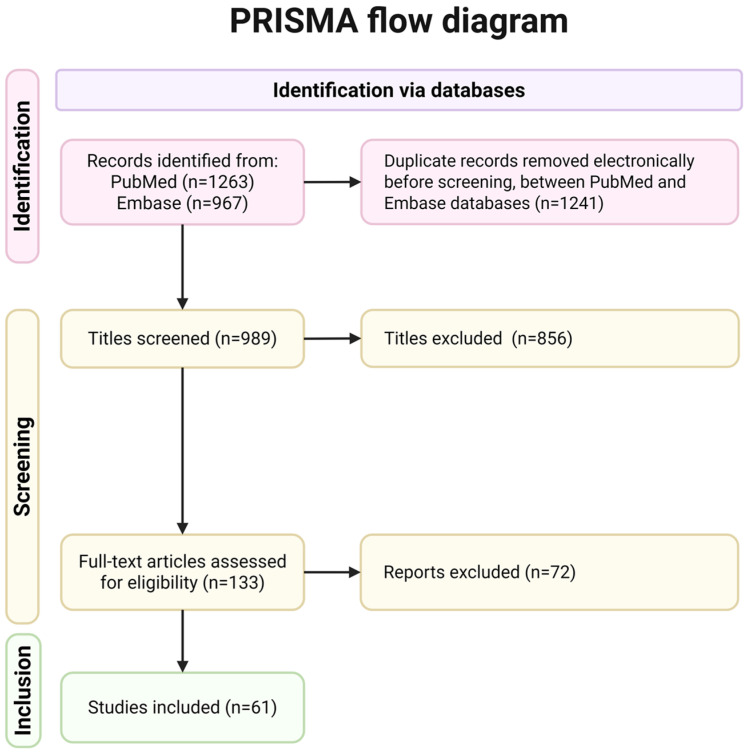
PRISMA flowchart showing the process of literature search and study selection.

We performed a screening of all the material, focusing on title and abstract, if they were linked with autoimmune endocrine diseases, and their genetic and epigenetic mechanism; they were then selected to be further analyzed. Articles that were duplicates, were not strict about autoimmune disease, or were not applicable to humans were ruled out. Further screening was performed based on the material being free. The selected material to be read was then further screened on the same basis as before and on their ability to provide a deeper look into the genetic mechanism (Figure 1).

## 3. Results

### 3.1. Autoimmune Thyroid Diseases (AITD)

Autoimmune thyroid diseases (AITD), encompassing Graves’ disease (GD) and Hashimoto’s thyroiditis (HT), are among the most common autoimmune disorders, affecting ~5% of the population [1]. Loss of immune tolerance underpins both entities but with distinct effector patterns. In GD, thyroidal T-cell infiltration and B-cell activation lead to the production of TSH-receptor autoantibodies (TRAb), whose binding to TSHR drives goiter, hyperthyroidism, and extrathyroidal manifestations such as ophthalmopathy and dermopathy. In HT, a predominantly cell-mediated response with high inflammatory load and apoptosis causes progressive thyroid tissue destruction and hypothyroidism; humoral autoimmunity frequently coexists, with anti-TPO and anti-Tg positivity [1,10]. The etiology of AITD is multifactorial: genetic factors explain a substantial share of susceptibility, while epigenetic and environmental modifiers contribute to phenotypic variation (Table 2).

#### 3.1.1. Genetic Mechanisms in AITD

AITD cluster in families, and quantitative data underscore strong heritability with cross-phenotype overlap. In GD, concordance has been reported at 35% in monozygotic (MZ) twins versus 3% in dizygotic (DZ) twins; in HT, concordance rates of 55% (MZ) and 0% (DZ) have been described, with concordance for autoantibodies also higher in MZ than DZ twins (e.g., TgAb 59% vs. 23%; TPOAb 47% vs. 29%) [12]. Family studies reveal elevated recurrence among first- and second-degree relatives; a total of 6.1% of first-degree relatives of GD probands have GD, 4.9% of first-degree relatives of HT probands have HT, and a Mendelian dominant pattern has been suggested for the tendency to develop thyroid autoantibodies [1]. Importantly, intrafamilial and intrapair variation is common: in MZ twins, the co-twin of a GD case may develop HT (and vice versa), illustrating shared genetic architecture with divergent clinical expression [1,12]. These data support a model in which shared susceptibility alleles are modulated by epigenetic and environmental inputs to determine phenotype.

Susceptibility genes fall broadly into (i) immune-regulatory loci—HLA-DR3, PTPN22, CD40, FOXP3, CTLA4—and (ii) thyroid-specific antigen genes—Tg, TPO, TSHR [1,10,11].

##### HLA Region

The MHC on 6p21 encodes HLA molecules that present peptides to T-cells, shaping central and peripheral tolerance. Polymorphisms influence peptide binding, thymic selection, and downstream immune programs [10]. HLA-DR3 (DRB1*03) shows a strong association with GD—~40–55% in Caucasian GD versus 15–30% in the general population—and contributes to shared susceptibility with type 1 diabetes [2,6]. Signals for HT are less consistent, but a predisposing effect is often suggested [10,14]. Graves ophthalmopathy (GO) and response to therapy have been linked to specific HLA patterns [15].

##### Costimulation and Lymphocyte Signaling (CTLA4, PTPN22, CD40, FOXP3)

**Figure 2:**
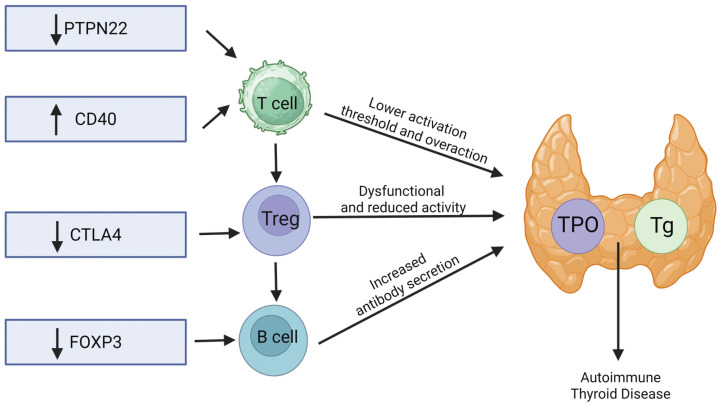
Thyroid autoimmune disease mechanisms.

PTPN22 (R620W, rs2476601) is a well-replicated non-HLA risk allele for AITD (Figure 2), with population-dependent effects [1,10,14]. CD40 promoter/UTR variants (e.g., rs1883832) influence expression and associate with GD, with distinct signals reported in HT [1]. CTLA4 variants (e.g., +49A/G; rs231775/rs231779) alter checkpoint function and have been repeatedly linked to GD and broader AITD phenotypes [1,10,14]. FOXP3 polymorphisms (e.g., rs3761548, rs3761549) may compromise Treg stability or function; meta-analyses report GD associations (ancestry-specific for rs3761548) and links to HT and TPOAb titers [1,13,15].

FOXP3 genetic variation has also been associated with clinical course—e.g., remission versus intractable GD and HT severity—suggesting prognostic value [1].

#### 3.1.2. Thyroid-Specific Susceptibility Genes

##### TSHR

TSHR (14q31) encodes the GPCR essential for thyroid growth and hormone synthesis and is a central autoantigen in GD, where TRAb mimics TSH signaling. Multiple intron-1 SNPs (notably rs179247, rs12101255) reproducibly associate with GD across cohorts and ancestries; additional variants influence ophthalmopathy risk [1,10,14]. The proposed mechanisms include altered alternative splicing in the thyroid (increasing soluble isoforms with greater immunogenicity) and modulation of thymic TSHR expression that shapes central tolerance [15].

TSHR variant patterns have been linked to ophthalmopathy, helping stratify prognosis for eye disease in GD [1,15].

##### TG

TG (8q24) encodes thyroglobulin, the iodinated precursor of T3/T4. Linkage/association studies across exons 10, 12, and 33 and intron 41 report haplotypes and SNPs associated with GD, HT, and TgAb positivity, with cohort-dependent effect sizes [1,10,14]. Structural changes to TG under conditions of high iodine may remodel processing and enhance the presentation of immunogenic peptides by HLA-DRβ-Arg74, increasing T-cell recognition [1,17].

##### TPO

TPO (2p25) encodes thyroid peroxidase. Several SNPs (e.g., rs2071400, rs2071403, rs732609) associate with TPOAb levels, HT risk, and disease course in selected cohorts, though the effects vary by ancestry and phenotype [1,10,14].

### 3.2. Epigenetics and AITD

Epigenetic remodeling integrates genetic risk with environmental input. In GD, global leukocyte hypomethylation is accompanied by pathway-specific changes, with many differentially methylated sites in intragenic regions; T-cell activation/signaling genes often display hypermethylation signatures [13,16,21]. The TSHR locus shows hypermethylation with reduced active histone marks in GD, consistent with regulatory control over transcript levels and splicing in the thyroid and thymus [16,20]. Epigenome-wide comparisons of GD and HT identify distinct DMPs/DMRs that persist after treatment and correlate with thyroid hormone levels, supporting disease-specific epigenetic architectures [20,21]. Age-related methylation trajectories at ATG4C, BECN1, and PRKAB1 further modulate AITD risk with aging [9]. From pre-clinical studies, histone-mark biology adds phenotype nuance: JMJD3/KDM6B (H3K27 demethylase) contributes to inflammatory stabilization in HT (pharmacologic GSK-J4 is modulatory) [22], whereas LSD1/KDM1A (H3K9 demethylase) promotes orbital adipogenesis in GD-associated ophthalmopathy [18].

Promoter DNA-methylation patterns have been associated with GD severity and treatment response, indicating potential prognostic utility [16,20].

### 3.3. Environmental Factors and AITD

Multiple exposures modulate risk, including smoking, alcohol, iodine status, infections, medications (e.g., interferon-α, amiodarone), stress, pregnancy/postpartum, selenium status, ionizing radiation, and features of the gut microbiome and broader exposome [10,20,23].

Excess iodine is a risk factor for AITD, plausibly by increasing TG antigenicity (e.g., reactive-oxygen-species–mediated structural changes) and amplifying autoimmune responses [1,19,21].

Infectious triggers—commonly assessed serologically—include Yersinia enterocolitica, Helicobacter pylori, Borrelia burgdorferi, hepatitis C virus, Hantavirus, Saccharomyces, Toxoplasma gondii, and HIV; differences in gut microbiota composition between GD and HT have been reported [1,23,24]. Therapy-related exposures are clinically relevant: interferon-α is strongly associated with thyroiditis in predisposed individuals [10,20].

### 3.4. Other Nongenetic and Nonnutritional Factors

Fetal–maternal microchimerism reflects bidirectional trafficking of cells during pregnancy, with long-term persistence in the host. Data on its role in AITD are conflicting, with studies reporting both higher and lower fetal cell counts in cases versus controls, suggesting context-dependent effects on tolerance and inflammation [10].

### 3.5. Autoimmune Pituitary Disease

**Figure 3:**
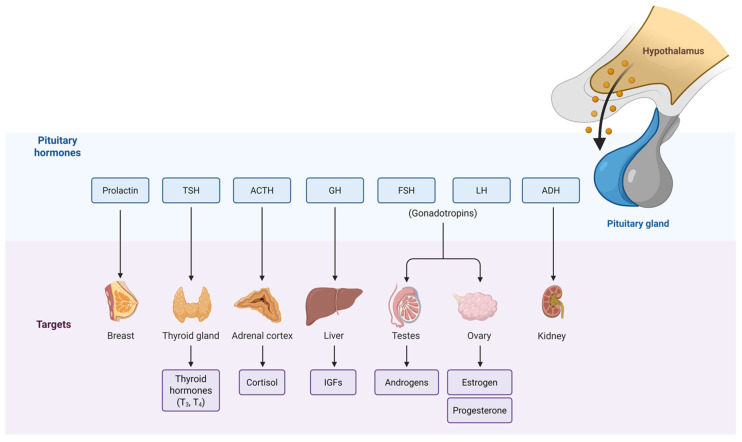
Hypothalamic–pituitary–peripheral action axis.

Hypopituitarism is a condition characterized by the reduced secretion of one or more of the hormones produced by the pituitary gland. Because the pituitary gland orchestrates endocrine axes that regulate growth, metabolism, reproduction, and stress responses, hypopituitarism disrupts systemic homeostasis and is linked to impaired quality of life and a worse prognosis; importantly, secondary adrenal insufficiency may precipitate life-threatening adrenal crisis (Figure 3). It is classically divided into congenital and acquired causes. Hypopituitarism is frequently accompanied by other systemic and localized diseases [9].

Congenital hypopituitarism often stems from disruptions in transcription factors and signaling pathways critical for pituitary organogenesis. Acquired hypopituitarism, frequently associated with pituitary tumors or systemic diseases, has also been increasingly linked to autoimmune mechanisms.

Hypophysitis is an inflammatory disorder of the pituitary that commonly presents with hypopituitarism. Clinically, corticotroph (ACTH) and thyrotroph (TSH) deficiencies are typical, with hypogonadism also frequent; diabetes insipidus and hyperprolactinemia may occur. By etiology, hypophysitis is classified as primary (idiopathic/autoimmune) or secondary (due to a systemic disease, local lesion, or drug) [9,25].

#### 3.5.1. Primary Hypophysitis

Primary hypophysitis is rare (around 1 per 9 million per year), and the definitive diagnosis requires histological confirmation; a probable diagnosis may include typical neuroimaging criteria (symmetricaly thickened pituitary stalk, homogeneous enhancement after contrast agents, dural tail sign, intact sellar floor, or pituitary shrinkage over time in chronic phase), any pituitary deficiency, and the exclusion of secondary causes such as tumors or infiltrative lesions. The histopathologic subtypes include lymphocytic (the most common), granulomatous, xanthogranulomatous, and necrotizing. Lymphocytic hypophysitis, otherwise known as autoimmune hypophysitis, can be anatomically subdivided into lymphocytic adenohypophysitis (LAH), lymphocytic infundibulo-neurohypophysitis (LINH), and lymphocytic panhypophysitis (LPH) [9].

Lymphocytic hypophysitis (LH) represents the most frequent form of hypophysitis, accounting for approximately two-thirds of all cases. LH predominantly affects women, particularly during the late stages of pregnancy or the immediate postpartum period, with a primary impact on the anterior pituitary. A common symptom of LH is adrenocorticotrophic hormone (ACTH) deficiency. Autoimmunity underpins primary disease, with genetic susceptibility (e.g., specific HLA backgrounds) and pituitary-directed autoantibodies reported (Table 3).

An autoimmune basis is supported by HLA associations—HLA-DQ8 and HLA-DR53—and by identified pituitary autoantigens and autoantibodies, including GH1/GH2, ENOL1, PGSF1a/PGSF2, SCG2, and lactotroph-targeting antibodies [9,26].

##### Genetics and Hypopituitarism

Hypophysitis is a disorder whose genetic basis is still not fully understood due to its complex etiology and the possible involvement of other causes of hypopituitarism, such as congenital diseases that lead to abnormal pituitary gland development. Patients carrying mutations in the POU1F1 or PROP1 genes may present with this disorder, making it necessary to differentiate child-onset hypopituitarism from pediatric hypophysitis and pituitary hyperplasia. Multiple studies report the presence of certain human leukocyte antigen (HLA) polymorphisms in patients with PAH, supporting an antigen-presentation-driven mechanism. For example, HLA-DR4 and HLA-DR5 were found in 44% and 23% of cases, respectively, between 1987 and 1999, and HLA haplotypes DQ8 and DR53 were identified in 87% and 80% of cases, respectively, in a series of 15 PAH patients. A recent study identified the presence of 12 HLA haplotypes associated with celiac disease in 16 consecutive Caucasian patients affected by PAH, with the prevalence of the DQ8 haplotype being 25%. Moreover, individuals carrying the DQ8 haplotype were four times more likely to develop PAH [9,25].

#### 3.5.2. Secondary Hypophysitis

Secondary hypophysitis results from systemic or local conditions, including Sjögren’s syndrome, systemic lupus erythematosus, sarcoidosis, Langerhans cell histiocytosis, and IgG4-related disease; it also includes drug-induced forms (e.g., immune checkpoint inhibitors targeting CTLA4 or PDCD1/PDCDL1, daclizumab) and rare post-vaccination cases (including reports linked to SARS-CoV-2 infection or vaccination) [25].

#### 3.5.3. ICI-Induced Hypophysitis

Hypophysitis is an increasingly frequent endocrine toxicity of clinical significance observed in patients on treatment with ICIs, particularly monoclonal antibodies (mAbs) that target cytotoxic T-lymphocyte antigen 4 (CTLA4) and programmed cell death-1 (PDCD1). Nonetheless, there are limited data available on the molecular mechanism underlying this disorder.

##### Genetic Factors and ICI-Induced Hypophysitis

The pathogenesis of IIHs involves various mechanisms, among which genetic factors play a crucial role.

It is well known that genetic polymorphisms of CTLA4 and PDCD1 genes can increase the risk of developing autoimmune diseases, including IIHs. These polymorphisms may not alter the CTLA4 amino acid sequence but can affect the affinity for CTLA4 mAbs, thereby increasing the risk of immunotherapy-induced autoimmune disorders.

Several HLA haplotypes have been shown to be associated. A Japanese study showed that HLACw12 and HLA-DR15 were significantly associated with anti-CTLA4-related hypophysitis, whereas HLA-DQB106:01, HLA-DPB109:01, and HLA-DRB5*01:02 were significantly associated with anti-PD-1-related hypophysitis. Another study found that HLA-Cw12, HLA-DR15, HLA-DQ7, and HLA-DPw9 were significantly more prevalent in patients with immunotherapy-induced central hypoadrenalism.

This HLA association presents a possible alternative mechanistic hypothesis in contrast to the proposed hypothesis that ICI hypophysitis is due to direct binding of CTLA4 inhibitors to pituitary cells.

Additionally, copy number variations (CNVs) and small variations (VARs) may also be associated with the occurrence of IIHs. A study analyzing 95 melanoma patients treated with ICIs found that genes affected by VARs associated with hypophysitis include SMAD3, PRDM1, and IL1RN, while genes affected by CNVs related to the occurrence of hypophysitis are TERT, SMAD3, JAK2, PRDM1, FAN1, CD274, and UNG [26].

#### 3.5.4. Anti-POUF1F1 Hypophysitis

Anti-POU1F1 hypophysitis (anti-POU1F1 antibody syndrome) is a newly described pituitary autoimmune disease characterized by acquired and specific growth hormone (GH), prolactin (PRL), and thyroid-stimulating hormone (TSH) deficiencies.

This disorder is associated with a thymoma or neoplasm that ectopically expresses pituitary-specific transcription factor 1 (POU1F1) protein. Circulating anti-POU1F1 antibody is a disease marker, and POU1F1-reactive cytotoxic T-cells (CTLs) play a pivotal role in disease development. Immunohistochemical analyses of pituitary tissue from autopsies revealed the persistence of ACTH-, LH-, and FSH-positive cells, while POU1F1-positive (GH, PRL, and TSH) cells were absent. Lymphocytes (rather than serum alone) are reactive to POU1F1, and tolerance appears to break via ectopic POU1F1 expression in tumors (classically thymoma but also other malignancies).

##### Genetic and Anti-POU1F1 Hypophysitis

The genetic background regulating the threshold of autoantibody production in B-cells has also been implicated. Studies established that processed POU1F1 epitopes are presented on the cell surfaces of anterior pituitary cells via major histocompatibility complex (MHC)/human leukocyte antigen (HLA) class I molecules [9].

With regard to thymoma, it has been reported that various genes associated with regulation are mutated in thymic carcinoma, and epigenetic abnormalities are common in thymoma. Given that many malignancies exhibit abnormal epigenetic regulation, it is plausible that these altered epigenetic modifications result in ectopic POU1F1 expression, and some studies are being conducted, even if no detected methylation abnormalities have been found in the regulatory region of the POU1F1 gene in thymoma [2]. Prospective, multi-center cohorts integrating germline HLA/immune-gene genotyping, tumor genomics, and paired blood/tumor epigenomics (methylome, chromatin accessibility) are needed to define causal pathways and develop predictive risk tools for autoimmune hypophysitis.

### 3.6. Autoimmune Parathyroid Disorders

Hypoparathyroidism is an endocrine disorder characterized by insufficient secretion of parathyroid hormone (PTH), presenting with hypocalcemia, hyperphosphatemia, and a relatively high urinary calcium excretion [27]. Autoimmune hypoparathyroidism is rare and may occur as an isolated, acquired disease or as part of autoimmune polyendocrinopathy syndromes (APS) [27,28]. It can be caused by permanent hypoparathyroidism, owing to irreversible, immune-mediated damage to the parathyroid glands, or functional hypoparathyroidism as a result of antibody-induced activation of signaling pathway(s) regulating parathyroid function [29].

In isolated cases, pathogenic mechanisms include activating autoantibodies against the calcium-sensing receptor (CaSR), which suppresses parathyroid hormone (PTH) secretion despite hypocalcemia [30], as shown in sporadic patients and in immune checkpoint inhibitor-induced hypoparathyroidism [29,31,32].

In APS-1, otherwise known as autoimmune polyendocrinopathy-candidiasis-ectodermal dystrophy (APECED), hypoparathyroidism represents one of the three major diagnostic criteria and occurs in 73–90% of patients, usually together with chronic mucocutaneous candidiasis and Addison’s disease [33,34]. Screening for anti-IFN-ω and anti-IL-22 autoantibodies provides highly sensitive diagnostic markers [4,5,28,34] often accompanied by anti-IL-17A/F; these cytokine autoantibodies are valuable for early recognition and risk stratification, while antibodies against NALP5 and CaSR may predict or accompany parathyroid failure [27]. Outside APS-1, autoimmune hypoparathyroidism can coexist with autoimmune thyroid disease (often catalogued as APS-3) or with diverse organ-specific autoimmunity (APS-4), and when no APS-1 hallmarks or disease-specific autoantibodies are detected, the autoimmune etiology rests on clinical clustering and exclusion of alternatives [27]. In the forms without specific autoantibodies (NALP5Abs, IFNωAbs, etc.) or AIRE gene mutations, the autoimmune etiology remains hypothetical (Table 4).

In such non-APS-1 HP, only the association with class I human leucocyte antigen (HLA) allele A*2601 and with class II HLA alleles DRB1*01 and DRB1*09 has been demonstrated [28,30].

#### 3.6.1. Genetic Mechanisms in Autoimmune Parathyroid Disease

The best defined genetic substrate of autoimmune parathyroid disease is biallelic loss-of-function mutations in AIRE [5], a transcriptional regulator that ensures central immune tolerance by promoting thymic expression of tissue-specific antigens [27]. More than 120 AIRE mutations have been described worldwide, with population-specific founder effects [33,34,35]:

- p.R257X in Finland and Eastern/Central Europe;
- p.R139X in Sardinia;
- p.Y85C in Iranian Jews;
- p.R203X in Southern Italy/Sicily;
- The 13 bp exon 8 deletion (p.Leu323fs) in British and North American cohorts [4,6,33].

Despite identical AIRE mutations, striking phenotypic variability occurs, even in siblings [38]. For example, in Finnish and Indian cohorts, patients homozygous for the same founder mutation presented with divergent severity, some showing early candidiasis and others predominately endocrine failure [4,5,38]. This variability highlights the modifying role of nongenetic factors. Rarely, isolated hypoparathyroidism due to AIRE mutations has been documented [30,34,39], with decades of follow-up in which no other APS-1 features developed (exome-sequencing series and kindreds) [39].

In addition to recessive inheritance, dominant-negative AIRE mutations have been reported, leading to milder or non-classical APS-1 with incomplete penetrance [36]. Indeed, the G228W mutation was discovered in a patient from Tuscany affected by CH and autoimmune thyroiditis with the presence of circulating 21OH antibodies [13]; whilst the c.932G>A (p.C311Y) AIRE variant was found in a North-African patient affected by CMC, AAD, enamel dystrophy, partial diabetes insipidus, and pernicious anemia [6]. Oftedal and colleagues identified heterozygous, dominantly acting AIRE missense variants clustering in the PHD1 zinc-finger domain, associated with later onset, milder autoimmunity and reduced penetrance (“non-classical” APS-1), whereas most recessive missense variants in classical APS-1 map to the N-terminal CARD/HSR region; thus, whether an AIRE allele behaves recessively or dominantly is strongly influenced by its position and the domain it perturbs [37]. They propose that APS-1 can be dived into (i) a “classical” recessive entity (≥2 core components and anti–type-I-IFN antibodies) and (ii) a “non-classical” entity due to dominant heterozygous PHD1 variants with milder, less penetrant autoimmunity [32].

Genotype–phenotype nuances support this view: in Iranian Jews, p.Y85C has been associated with lower rates of chronic mucocutaneous candidiasis (CMC) and Addison disease; most Finnish carriers of p.R257X manifest CMC; exon-8 c.967–979del13bp cohorts showed less CMC but more alopecia in some European series; whereas an Indian APECED series homozygous for c.967–979del13bp exhibited frequent CMC (~75%) and relatively infrequent alopecia (~17%), underscoring population modifiers [5].

Beyond AIRE, immune effectors also play a genetic/immunologic role. Autoantibodies against CaSR can act as functional receptor agonists, directly inhibiting PTH secretion and causing hypocalcemia [29,31,32]. Similarly, NALP5 autoantibodies have been associated with parathyroid autoimmunity in APS-1 [34]. Furthermore, HLA associations support an adaptive immune contribution: HLA-A*26:01 (MHC class I) is strongly linked to idiopathic hypoparathyroidism, implicating CD8+ cytotoxic T-cells, while HLA-DRB1*01 and HLA-DRB1*09 alleles are enriched in affected patients [27].

#### 3.6.2. Epigenetics and Hypoparathyroidism

Although direct epigenetic studies in autoimmune hypoparathyroidism are limited, AIRE function is inherently chromatin dependent. AIRE induces transcription of tissue-specific antigens at loci with a repressive chromatin state, promoting the deletion of autoreactive T-cells and the development of regulatory T-cells (Cranston et al., 2022 [34]). AIRE also exerts TSA-independent functions relevant to central tolerance, including modulation of chemokine expression, regulation of mTEC apoptosis and differentiation, and control of thymocyte migration [27].

Post-transcriptional regulation has been implicated as well: miR-220b has been shown to directly regulate AIRE expression, linking microRNA dysregulation to defective immune tolerance [37]. Other transcriptional regulators such as Fezf2 can independently orchestrate TSA expression programs, providing an additional chromatin-based mechanism that cooperates with or compensates for AIRE [37].

Evidence from clinical cohorts further supports an epigenetic/environmental contribution. In Indian and Finnish series, siblings with identical AIRE mutations often displayed markedly different clinical courses [5,36]. Similarly, microbiota differences across populations may contribute to variability in autoimmune phenotype despite shared genotypes [33]. Moreover, sex-specific penetrance of hypoparathyroidism in APS-1 underscores the likely role of epigenetic or hormonal modifiers [27]. Taken together, these findings indicate that, while APS-1 is a monogenic disorder, its clinical expression reflects a complex interplay between genetics, epigenetics, and environment [39].

Of note, sex differences exist: penetrance of hypoparathyroidism in APS-1 is nearly 100% in women but <60% in men, while Addison’s disease shows no sex bias [27,38]. This discrepancy suggests hormonal or epigenetic modifiers influence the expression of parathyroid autoimmunity.

Collectively, these data reinforce that AIRE’s role in parathyroid and broader organ-specific autoimmunity is not binary (present/absent) but gradated by the affected domain, allelic behavior (recessive vs. dominant-negative), and modifying factors (sex, environment, microbiome).

### 3.7. Autoimmune Adrenal Disease

Adrenal insufficiency (AI) denotes impaired glucocorticoid production due to reduced adrenal function. Three etiologic categories are recognized: primary AI (PAI) due to adrenal pathology; secondary AI from deficient pituitary ACTH; and tertiary AI from hypothalamic dysfunction (often grouped with secondary AI). Primary AI, known as Addison’s disease (AD), is rare, with a prevalence of 100 per million in Europe, and results from damage or malfunction of the adrenal cortex leading to deficient cortisol, aldosterone, and DHEA synthesis. In autoimmune AD (often termed autoimmune Addison disease, AAD), a consistent serological hallmark is the presence of 21-hydroxylase (CYP21A2) autoantibodies, detectable in ~85% of patients near diagnosis. By contrast, secondary AI reflects inadequate ACTH drive with consequent glucocorticoid deficiency and relatively preserved mineralocorticoid production [7,8].

#### 3.7.1. Addison’s Disease

**Figure 4:**
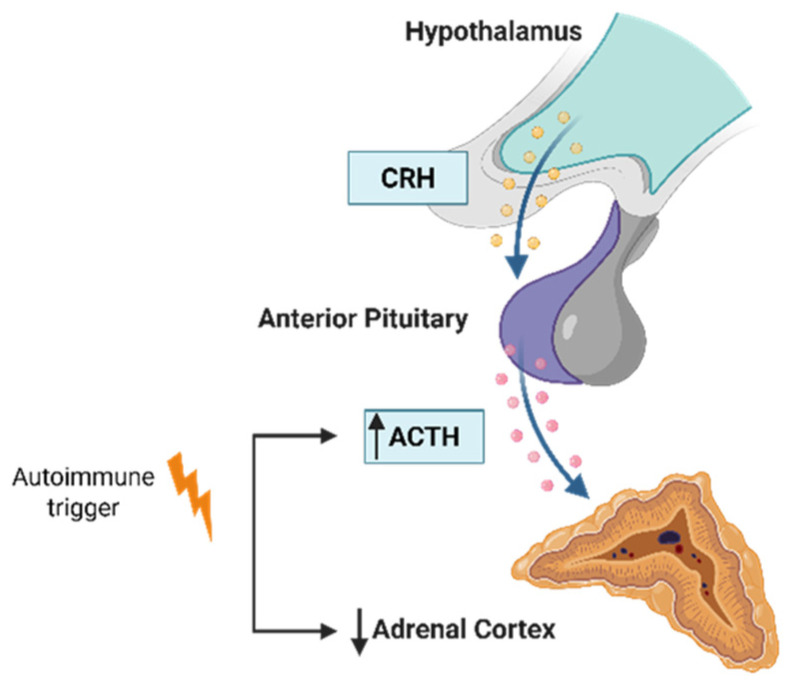
Adrenal autoimmunity mechanism.

Autoimmune destruction of the adrenal cortex is the most common cause of PAI. Pathogenesis is multifactorial, with polygenic genetic susceptibility and environmental modifiers (Figure 4). AAD may occur in isolation or, in a substantial fraction, co-aggregate within autoimmune polyglandular syndromes (APS)—particularly APS-2 but can also present in the context of APS-1 due to AIRE mutations (Table 5) [7,8]. Large population analyses (including a UK cohort exceeding 22 million individuals) indicate that AAD risk clusters with many other autoimmune diseases, consistent with shared genetic architecture and, in some settings, iatrogenic influences from immunomodulatory therapies used for coexisting autoimmunity [37,40].

##### Addison’s Disease and Genetic Mechanisms

Except for monogenic APS-1, most AAD does not follow Mendelian inheritance; nevertheless, familial clustering and twin data support high heritability [7,8]. Candidate gene studies established HLA class II as the dominant susceptibility region, particularly DR3–DQ2 and DR4–DQ8 haplotypes. Additional immune-regulatory loci include MICA/MICB, CTLA4, PTPN22, CIITA, CLEC16A, CD274 (PD-L1), and NLRP1 (NALP1)—variants shared with other autoimmune diseases, underscoring common immune checkpoints rather than AAD-specific genes. A disease-specific AAD locus has not been definitively identified, although HLA-DRB1*04:04 has been implicated in some populations [8]. Beyond common variants, rare variants in immune genes such as RAG1, TNFAIP3 (A20), LAT, and IKZF2 (HELIOS) have been described in autoimmune syndromes that include adrenal autoimmunity [8].

The first genome-wide association study of AAD (1200 cases; Wolff et al. [7]) reported nine risk loci, collectively explaining 40% of common-variant risk, with the HLA region contributing the largest effect. Previously implicated genes (BACH2, PTPN22, CTLA4) were confirmed, and additional loci (LPP, SH2B3, SIGLEC5, UBASH3A) were identified. Notably, AIRE showed common-variant associations, indicating that AIRE contributes to risk both in monogenic APS-1 and in the polygenic spectrum. Together with AIRE signals in type 1 diabetes and pernicious anemia, these data support an AIRE dosage/functional-level effect on organ-specific autoimmunity [7,8]. In Swedish AAD, polygenic risk modeling (Røyrvik et al.) showed that each additional risk allele more than doubled the odds of disease and advanced age at onset by >8 years when comparing individuals with >9 versus <5 risk alleles; a polygenic risk score (PRS) distinguished typical autoimmune PAI from pediatric monogenic PAI, in which PRS values were clearly lower—suggesting clinical utility for triage toward genetic testing when PRS is low [8].

Copy-Number Variation (CNV)

A CNV study (Brønstad et al.) identified two loci associated with AAD near UGT2B28 (chromosome 4) and ADAM3A (chromosome 8) [3]. Although mechanisms remain to be clarified, UGT2B28 participates in steroid glucuronidation (implicating altered steroid inactivation), and ADAM-family genes influence inflammatory signaling and T-cell biology (suggesting effects on immune maturation). The UGT2B28 single-copy state (CN = 1) was enriched in cases but is also relatively common in the general population, and the locus overlaps the UGT2B17 region, so specificity for autoimmunity remains uncertain. These data highlight that gene dosage (copy number) may add to sequence variation in shaping AAD risk [3].

##### Addison’s Disease and Epigenetic Mechanisms

Because AAD is strongly associated with HLA class II, CD4^+^ T-cell-mediated processes are central. Epigenetic profiling in CD4^+^ T-cells has reported global hypomethylation in AAD relative to controls, with a majority of differentially methylated regions (DMRs) in promoter elements and an overall skew toward hypomethylation; these patterns are comparable to those observed across other organ-specific autoimmune diseases and may reflect persistent activation states in disease-relevant pathways [8].

#### 3.7.2. Autoimmune Polyendocrine Syndrome (APS)

APS-1 (APECED) is a rare, typically childhood-onset disorder caused by biallelic pathogenic variants in AIRE, resulting in defective central tolerance. Classically, diagnosis rests on two of three major features: primary adrenal insufficiency, hypoparathyroidism, and chronic mucocutaneous candidiasis, although numerous minor components occur and clinical expressivity is broad [33,35,37]. The human AIRE gene (21q22.3) spans 14 exons encoding a 545 aa protein with a CARD/HSR region (oligomerization), SAND domain, LXXLL motifs, and two PHD zinc fingers that coordinate co-factor recruitment and chromatin engagement. More than 100 pathogenic variants have been reported; many alter splicing, producing exon-skipped or intron-retained transcripts with reduced transcriptional activity. Founder effects contribute to geographic clustering (e.g., p.R257X in Finland and parts of Central/Eastern Europe; p.R139X in Sardinia; p.Y85C in Iranian Jews; recurrent 13 bp exon 8 deletions in British/Irish/North American cohorts) [30,33,36]. Although robust genotype–phenotype rules are elusive, dose–response between residual AIRE function and disease severity is emerging: dominant-negative or hypomorphic alleles can cause non-classical APS-1 with later onset and reduced penetrance [36]. Beyond monogenic APS-1, common AIRE variation (e.g., p.R471C) contributes to polygenic risk for Addison’s disease, pernicious anemia, and type 1 diabetes, reinforcing the concept that AIRE dosage influences a continuum from rare to common autoimmunity [7,8].

Autoantibodies are a defining feature of APS-1. Neutralizing anti–type I interferon (IFN-α subtypes and IFN-ω) antibodies are highly specific, often present before clinical disease, and now serve as early diagnostic biomarkers; other antibodies (e.g., anti–IL-17/IL-22) explain susceptibility to candidiasis, while organ-specific targets (e.g., NALP5 in APS-1 hypoparathyroidism) can assist phenotypic classification and monitoring [33,35,37]. Importantly, non-endocrine manifestations may precede the classic triad; incorporating such features into diagnostic criteria improves early detection and care trajectories [33].

### 3.8. Autoimmune Gonadal Disease

Autoimmunity connected to gonadal failure, namely autoimmune oophoritis in females and autoimmune orchitis in males, arises when immune tolerance to gonadal antigens breaks down, leading to inflammatory damage of hormone-producing and gametogenic tissues. The clinical consequence is frequently subfertility or infertility [40]. Risk is heightened in patients with coexisting endocrine (e.g., Hashimoto’s thyroiditis, Graves’ disease, autoimmune polyglandular syndromes) and non-endocrine autoimmune diseases due to underlying mechanisms of genetic predisposition, dysregulated immune responses, and environmental triggers (Table 6) [40]. Genetic predisposition plays a significant role; a prominent example is mainly linked to autoimmune regulator (AIRE) gene mutations as the AIRE gene is crucial for maintaining self-tolerance by preventing T-cell attacks on the body’s tissues [41].

#### 3.8.1. Autoimmune Testicular Disease

The testis is considered an immunoprivileged organ: the blood–testis barrier, specialized Sertoli–germ cell junctions, local immunoregulatory mediators, and constrained antigen presentation together protect from immune attack germ cells that appear after puberty when immunocompetence is already established [56]. Nonetheless, infection, trauma, surgery, or congenital anomalies can expose sperm antigens to the immune system and precipitate anti-sperm antibodies (ASAs). ASAs are detectable in ~5–12% of men evaluated for infertility and can bind sperm in serum or seminal plasma, impairing motility and fertilizing capacity. When testicular immune privilege is breached and immune regulation fails to re-establish tolerance, a sustained autoimmune orchitis may ensue, characterized by testicular inflammation with ASAs in the absence of an infectious origin [3].

While environmental triggers such as infection or trauma may initiate inflammation, genetic predisposition is the primary determinant of whether a chronic autoimmune response develops. Evidence from human monogenic disease highlights both central tolerance defects and peripheral regulation of antigen presentation as key genetic mechanisms [41,42].

##### Genetic Mechanisms

The best characterized genetic cause is the AIRE gene. AIRE encodes a transcriptional regulator that establishes central tolerance by driving thymic expression of tissue-restricted antigens, including those from the male reproductive tract [41]. Inactivating mutations of AIRE causes autoimmune polyendocrine syndrome type 1 (APS-1), a monogenic disorder in which 12–30% of males develop primary hypogonadism with anti-sperm autoantibodies [42,43] In *Aire*-deficient mice, infertility is multifactorial: males show impaired mating, reduced testosterone, defective sperm fertilization, and autoimmune infiltration of the testis, epididymis, and prostate [42]. Autoreactivity can be directed to male reproductive proteins such as transglutaminase-4 (TGM4) and seminal vesicle secretory protein-2 (SVS2), underscoring that AIRE-dependent thymic display of reproductive antigens is central to preventing testicular autoimmunity [41].

Finally, MHC gene variants themselves remain the strongest polygenic contributors to autoimmune disease risk [3,7]. Certain MHC class II alleles present testis-specific antigens more effectively, favoring the activation of autoreactive T-cells and the development of orchitis. The role of MHC thus operates both directly, through allele-specific antigen presentation, and indirectly.

##### Epigenetic Mechanisms

Direct studies of epigenetic regulation in autoimmune male gonadal disease are lacking; however, AIRE itself functions through an epigenetic mechanism: it binds to non-methylated histone H3K4 (H3K4me0) and recruits transcriptional machinery to otherwise silent loci, enabling expression of testis-restricted antigens in the thymus [41]. In the absence of this chromatin-based control, autoreactive T-cells escape deletion and drive autoimmune orchitis. Beyond AIRE, no data currently implicate DNA methylation, histone modification, or non-coding RNAs in autoimmune orchitis specifically, highlighting an important research gap.

#### 3.8.2. Autoimmune Ovarian Disease

The ovary is not an immunologically privileged organ, but a breakdown in tolerogenic mechanisms for ovary-specific antigens has disastrous consequences on fertility in women. Primary ovarian insufficiency (POI) denotes loss of ovarian function before age 40 and typically presents with persistent oligo/amenorrhea, menopausal-range gonadotropins, and low estradiol [40]. A total of 20–30% of cases show autoimmune involvement, manifesting as an isolated ovarian disorder, in association with other organ-specific autoimmunity—most commonly autoimmune thyroid disease or Addison disease—or within autoimmune polyglandular syndromes (APS) [44,45]. Serologic markers (anti-ovarian or steroidogenic-cell antibodies) are variably positive and lack sufficient sensitivity and specificity for routine diagnosis; ovarian biopsy is discouraged because of low diagnostic yield and the risk of depleting residual follicles. Across series, autoimmune involvement is reported in roughly one-fifth to one-third of POI, and co-aggregation with thyroid and adrenal autoimmunity is frequent, underscoring shared susceptibility across endocrine organs [45].

A particularly important clinical interface is APS. When adrenal autoimmunity is present—typically signaled by steroidogenic-cell or 21-hydroxylase antibodies—histological autoimmune oophoritis is well documented; conversely, histological oophoritis is uncommon in the absence of adrenal autoimmunity. Additional ovarian antigens such as MATER/NLRP5 further support an ovary-targeted autoimmune endotype in a subset. In parallel, thyroid autoimmunity co-aggregates with ovarian hypofunction more often than expected by chance, reinforcing the view that ovarian and thyroid disease frequently express a common susceptibility architecture [46].

##### Genetic Mechanisms of POI

The most direct genetic route to autoimmune ovarian disease is failure of central tolerance. In APS-1, loss-of-function variants in AIRE impair thymic expression of tissue-restricted antigens, weaken negative selection, and permit survival of autoreactive T-cells; ovarian autoimmunity and infertility are well-recognized components, making AIRE the clearest germline determinant linking tolerance defects to ovarian failure [41].

Beyond this rare monogenic context, available evidence supports a polygenic liability shared with other autoimmune disorders: a bidirectional, two-sample Mendelian randomization analysis reported genetic links between autoimmune diseases and POI, consistent with distributed risk across immune checkpoints and lymphocyte-signaling pathways that can manifest in different endocrine targets, including ovary and thyroid [47]. Genomic background outside classical immune genes also modulates phenotype. High-resolution mapping reveals copy-number variation on the X chromosome enriched among women with POI; although not specific to the autoimmune subset, dosage effects in meiosis, DNA repair, and oocyte-maintenance genes plausibly reduce functional ovarian reserve and lower the threshold at which immune-mediated injury produces clinical failure [48]. Immune profiling in POI that shows diminished FOXP3^+^ regulatory T-cell tone and Th17 skewing is compatible with inherited tolerance fragility and aligns with the frequent coexistence of other autoimmune diseases or APS [44,49].

The thyroid–ovarian connection exemplifies this shared genetic architecture in practice: women with autoimmune thyroid disease have higher rates of menstrual disturbance, subfertility, diminished ovarian response, and POI, and women with autoimmune POI frequently carry thyroid autoimmunity. Indeed, HLA-DR3 haplotype is associated with increased risk of POF. This reciprocity argues for two-way clinical surveillance at diagnosis and follow-up [50].

##### Epigenetics of POI

Genetic predisposition alone does not explain why some women develop isolated ovarian disease, others evolve into APS, and still others manifest a thyroid–ovary dyad. Epigenetic programs (DNA methylation, histone modifications, and non-coding RNAs) translate genetic susceptibility and environmental cues into durable transcriptional states in immune and ovarian compartments, shaping penetrance, organ selectivity, and clinical course. Three complementary axes emerge consistently from the corpus provided.

First is a microRNA-tolerance checkpoint centered on microRNA-21 (miR-21) and Pellino-1 (Peli1). In patients with autoimmune POI, miR-21 and its target Peli1, an E3 ligase that restrains T-cell activation via MAPK and NF-κB, are concomitantly down-regulated. Lower levels correlate with reduced ovarian reserve (lower AMH and estradiol; smaller ovarian and uterine size), higher gonadotropins, and greater autoantibody burden. These observations place the miR-21–Peli1 axis at the interface of systemic autoimmunity and ovarian failure and cohere with reports of reduced regulatory T-cell tone in POI [51,52].

Second is an ovarian-intrinsic survival and steroidogenesis module governed by miR-21, MSX1, and Notch signaling. In ZP3-induced autoimmune POI, bone marrow mesenchymal-stem-cell exosomes enriched in miR-21-5p restore ovarian structure and function by directly repressing MSX1, a transcriptional brake on Notch. Relief of MSX1 repression activates Notch signaling, reduces granulosa-cell apoptosis (with decreases in p53 and Bax and an increase in Bcl-2), enhances proliferation, and up-regulates steroidogenic genes (FSHR, CYP11A1, CYP19A1), with concordant endocrine improvement (higher estradiol and AMH, lower FSH) and higher follicle counts. This pathway provides an ovarian counterpart to the tolerance module and explains how the same regulatory RNA can couple immune tone to granulosa-cell fate and steroidogenesis [53].

Third is an inflammatory–epigenetic circuit centered on NF-κB-primed NLRP3 inflammasome activation and pyroptosis. In autoimmune POI models (for example, IFN-γ injury and ZP3 immunization), aberrant NF-κB signaling primes NLRP3–caspase-1–GSDMD-mediated granulosa-cell pyroptosis. Mesenchymal-stromal-cell-derived exosomes suppress NF-κB and NLRP3, reduce pyroptosis, restore endocrine function, improve folliculogenesis, and, in vivo, enhance fertility—highlighting a tractable chromatin-linked inflammatory axis in autoimmune POI [54].

## 4. Discussion

Endocrine autoimmunity involves mainly T-cells, including CD4^+^ helper cells, such as Th1, Th17, and Tfh, that drive inflammation and support B-cell activation as well as CD8^+^ cytotoxic T-cells that directly kill endocrine cells [55]. Regulatory T-cells normally suppress autoreactive responses, and their dysfunction allows autoimmunity to develop. B-cells produce autoantibodies and present antigens to T-cells, sustaining the immune attack. Antigen-presenting cells, like dendritic cells and macrophages, initiate and amplify responses, while stressed or hyper-secreting endocrine cells can contribute to their own recognition by the immune system. Other cells such as stromal cells, endothelial cells, and natural killer cells also support inflammation and tissue damage.

Endocrine autoimmune diseases (EADs) result from complex interactions between genetic susceptibility and environmental triggers. The intricate relationship between infectious pathogens and autoimmunity has long been a central question in immunology. While infections can act as triggers of autoimmune processes, they may also confer protection against them, reflecting a dual, context-dependent influence [57]. Pathogen exposure thus represents a double-edged sword: depending on timing, genetic background, and the immune context, it can either initiate or suppress autoimmune responses. Among pathogens, *Staphylococcus aureus* hemolysin has been shown to influence the epigenome of Th1 and Th17 cells—key players in autoimmune pathogenesis—through alterations in gene expression and changes in DNA methylation [58,59,60].

Endocrine organs operate feedback loops controlling proliferation and secretion. Mutant cells that over-sense signals may hyper-proliferate and hyper-secrete, threatening homeostasis. To counter this, autoreactive T-cells may preferentially eliminate such aberrant cells—but this surveillance comes with a trade-off: a fragility to autoimmune disease [61].

The neuroendocrine immune system also plays an important role in several systemic diseases, including autoimmune disorders. Among the major players of NEI pathways are steroid hormones of the adrenal (cortisol) and gonadal glands (sex hormones), neurohormones such as melatonin, and, more recently, the vitamin D endocrine system [62].

Although there is increasing knowledge about autoimmunity mechanisms, in many endocrine autoimmune disorders, the standard of care is still hormone replacement rather than therapies addressing the autoimmune mechanism [63].

Across the endocrine axes examined, the literature supports a model in which shared immune-regulatory genetics (dominant HLA class II signals with reproducible contributions from *CTLA4* and *PTPN22*) interact with organ-proximal antigenic loci and epigenetic programs to produce distinct clinical phenotypes. In the thyroid, this framework is particularly well developed: immune-checkpoint loci converge with thyroid-specific genes (*TSHR*, *TG*, *TPO*), helping explain why susceptibility clusters within families yet partitions into Graves’ disease (GD) or Hashimoto’s thyroiditis (HT) depending on additional modifiers [1,2,3,6,10,11,14,15,16]. Family and twin evidence shows high heritability for both GD and HT, antibody concordance in monozygotic pairs, and occasional cross-phenotype switching, consistent with shared background risk and variable expression over time [1,12].

Epigenetically, global hypomethylation in GD coexists with locus- and pathway-specific changes, including hypermethylation/reduced active histone marks at *TSHR* and distinct DMP/DMR signatures separating GD from HT—even persisting after treatment, which argues that epigenetic state is not merely a consequence of thyroid status [16,20,21,22,52]. Several reports connect promoter methylation with disease severity and response in GD, suggesting emerging prognostic utility that warrants prospective validation [16,20,64]. Environmental inputs appear to be routed through these programs: excess iodine augments *TG* antigenicity and associates with methylation shifts in autophagy and signaling genes; infections (e.g., hepatitis C) and drugs (interferon-α, amiodarone) further lower the threshold for clinical disease [1,17,19,21]. The microbiome differs between GD and HT, but causality remains unsettled [23]. Together, these data support that epigenetic machinery encodes environmental exposures on a genetically primed immune system, shaping phenotype and course [13,15,16,17,19,20,21,23,24,65].

In hypophysitis, idiopathic/primary forms show HLA associations and candidate pituitary autoantigens, whereas ICI-induced hypophysitis highlights a live controversy: is toxicity driven by direct anti-CTLA-4 binding to pituitary cells or by host immunogenetic predisposition (specific HLA haplotypes and immune-gene variation) that primes the axis for injury under checkpoint blockade? Both mechanisms have support; limited multi-omic integration currently constrains inference. Reported VAR/CNV signals (e.g., TERT, CD274 (PD-L1), JAK2) and HLA associations with PD-1- or CTLA-4-related hypophysitis suggest that a host–tumor–drug triad—rather than a single pathway—drives risk, underscoring the need for HLA/immunogenetic + tumor genomic models to enable pre-therapy stratification [20,22,26].

For autoimmune hypoparathyroidism, APS-1 (biallelic AIRE defects) is the canonical central-tolerance failure, but agonistic anti-CaSR and anti-NALP5 antibodies explain isolated/APS-like phenotypes outside classic APS-1. Founder mutations and dominant-negative AIRE variants expand the clinical spectrum to “non-classical” APS-1 with lower penetrance, and striking intra-family variability argues for epigenetic and environmental modulation (and probable sex effects) in organ targeting and timing [27,28,29,30,31,32,33,34,35,36,37]. Neutralizing anti-type-I IFN antibodies are highly specific early biomarkers that improve recognition and risk stratification, illustrating how serology can shorten time to diagnosis beyond the traditional triad [33,35].

In autoimmune Addison’s disease (AAD), candidate-gene and GWAS data converge on HLA-DR3/DR4 with replicated non-HLA signals (CTLA4, PTPN22, BACH2, SH2B3) and common AIRE effects, extending an “AIRE dosage” concept from rare monogenic APS-1 into common, complex autoimmunity. Copy-number variation near UGT2B28 and ADAM3A suggests an added contribution from gene dosage, though mechanisms need clarification. Notably, polygenic risk scores (PRS) already separate typical autoimmune PAI from monogenic pediatric PAI and correlate with age at onset, providing a tractable route to triage genetic testing and to focus surveillance on high-risk carriers [3,7,8,40,41,42,43,46,56].

Gonadal autoimmunity illustrates the clinical reach of central tolerance pathways: AIRE deficiency perturbs reproductive-tract immune homeostasis (male infertility in Aire-null mice; human associations with testicular/epididymal pathology), while in women, a substantial subset of POI shows immune signatures (Th17 skewing; microRNA changes). Early-stage interventional studies (e.g., MSC-derived exosomes delivering miR-21-5p, repression of MSX1, suppression of NF-κB→NLRP3 pyroptosis) are mechanistically coherent but remain pre-clinical; translation will require robust human trials. Epidemiologic reciprocity between thyroid autoimmunity and ovarian hypofunction reinforces the value of two-way screening across these axes [44,45,46,47,48,49,50,51,52,53,54].

Clinical implications are as follows. (i) Adopt cross-axis surveillance: in AAD/APS-1, screen thyroid/ovary/parathyroid; in AITD, consider adrenal/ovarian autoimmunity. (ii) Use biomarkers with diagnostic and prognostic leverage: 21-hydroxylase for AAD; neutralizing anti-IFN for APS-1; anti-CaSR for unexplained hypocalcemia; DNAm signatures for risk stratification in AITD (pending validation). (iii) Manage iodine exposure prudently in genetically susceptible populations from iodine fasting areas. (iv) For ICIs, pilot HLA/PRS-based pre-treatment risk tools to personalize monitoring and consent. Limitations of the evidence include small or ancestry-restricted cohorts, cross-sectional epigenetics, heterogeneous phenotyping, and retrospective exposure assessment; these temper causal inference and generalizability.

Future directions include (1) longitudinal, multi-omic cohorts (germline, cell-type–resolved epigenome, transcriptome, microbiome) with harmonized outcomes; (2) external validation and clinical calibration of PRS (AAD) and HLA/immunogenetic panels (ICI-hypophysitis); (3) pragmatic trials of exposure modification (iodine, selenium) with molecular readouts; (4) early-phase studies of epigenetic-targeted strategies (e.g., JMJD3 and LSD1 modulators) in carefully phenotyped subsets; and (5) international standardization of biomarker panels (anti-IFN, anti-CaSR, NALP5) to unify screening and follow-up.

## 5. Conclusions

Endocrine autoimmunity reflects the intersection of shared immune genetics (HLA, *CTLA4*, *PTPN22*, *AIRE*), organ-specific antigen biology, and epigenetic/environmental context. This model accounts for co-aggregation across glands, intra-family heterogeneity, and GD↔HT switching. Translation to practice is already feasible in parts of the field—biomarker-guided diagnosis (anti-IFN, 21-OH, anti-CaSR), cross-axis screening, and early PRS-informed triage—while epigenetic and microRNA circuits offer promising but still experimental therapeutic footholds. Strategic investment in prospective, multi-omic, multi-ancestry research should convert these mechanistic insights into reliable prediction, prevention, and personalized treatment.

## Figures and Tables

**Table 1:** Susceptibility loci involved in endocrine autoimmune disease.

Locus (Lead Gene/Region)	Brief Function	Reported Disease Association(s)
HLA region (*HLA-DRB1*)	Antigen presentation (MHC class II)	GD, HT, AD (major genetic determinant across endocrine autoimmunity)
*HLA-DQA1*	MHC class II peptide binding	AITD (GD/HT)
*HLA-DQB1*	MHC class II peptide binding	AITD
*PTPN22* (LYP)	Tyrosine phosphatase regulating T-cell signaling	GD, HT, AD
*CTLA4* (CD125)	Immune checkpoint—negative regulator of T-cells	GD, HT, AD
*IL2RA* (CD25)	IL-2 receptor α chain—Treg biology	AITD
*SH2B3* (LNK)	Regulator of cytokine signaling/lymphocyte activation	several autoimmune diseases
*BACH2*	Transcription factor important for B/T-cell differentiation and tolerance	AITD (and other autoimmune diseases)
*CLEC16A*	Endosomal/autoantigen processing; immune regulation	AITD
*UBASH3A*	Negative regulator of T-cell receptor signaling	AITD
*CD226* (DNAM1)	Costimulatory receptor on T/NK-cells	GD
*RGS1*	Regulator of G-protein signaling; lymphocyte migration	AITD
*TNFAIP3* (A20)	NF-κB negative regulator; inflammation control	GD, HT, other autoimmune diseases
*STAT4*	Transcription factor downstream of IL-12/IFN signaling; Th1 responses	GD, HT
*AIRE*	Central tolerance (thymic expression of tissue antigens)	AD, APS (monogenic APS1); variant associations in AD GWAS
*FOXP3*	Master regulator of regulatory T-cells (Tregs)	APS/monogenic immune dysregulation; candidate associations in AITD
*TSHR*	Thyroid autoantigen/receptor	GD (major thyroid-specific locus)
*TPO*	Thyroid autoantigen (TPO)	HT/AITD
*TG*	Thyroid autoantigen (TG)	AITD (HT/GD)
*CD40*	B-cell costimulation; antigen presentation	GD, AITD
*VDR*	Immune modulation and endocrine signaling	Reported associations in GD/HT
*FCRL3*	B-cell Fc receptor-like protein; immune regulation	GD, AITD
*RNASET2*	Endonuclease; implicated by GD GWAS region (6q27)	GD (reported locus)
*IL2–IL21* locus (4q27)	Cytokines; T-cell growth (IL-2) and B-cell help (IL-21)	AITD
*IL10*	Anti-inflammatory cytokine (regulates immune tolerance)	AITD
*IL7R*	Lymphocyte survival and homeostasis	Autoimmunity (including T1D in some studies)
*RASGRP1*	T-cell receptor signaling cascade	AITD (reported)
*SPATA13*	Guanine nucleotide exchange factor; implicated in AITD transcriptome studies	AITD (putative association)
*CD247* (CD3ζ)	TCR complex signaling subunit	AITD (TWAS/expression)
*NKX2-3*	Homeobox TF; tissue/immune effects	AITD (reported in transcriptome studies)
*PDE8B*	cAMP metabolism; thyroid function regulation	AITD (expression/GWAS links to thyroid traits)
*CIP2A*	Cell growth regulator; implicated in AITD TWAS	AITD (reported)
*KIAA1109* 4q27	Immune-regulatory region near IL2/IL21	AITD, other autoimmune diseases
*DNASE1L3*	DNA nuclease linked to autoantigen clearance	Autoimmunity signals across disorders (reported)
*CD28/CTLA4*	Costimulation and checkpoint region	AITD
*PRDM1* (BLIMP1)	B-cell differentiation regulator; immune tolerance	Autoimmune disease associations
*IRF5*	Interferon regulatory TF; innate immunity	Broad autoimmunity (reported in composite studies)
*IRF8*	Myeloid/lymphoid TF; immune regulation	Autoimmunity cross-loci (reported)
*PTPRC* (CD45)	Phosphatase on leukocytes; T/B signaling	Autoimmune associations in multiple studies
*CCR6*	Chemokine receptor; lymphocyte trafficking	Reported in several autoimmune disease scans
*CCR5*	Chemokine receptor; immune cell migration	Reported candidate associations in endocrine autoimmunity
*CD40LG*	T-cell help to B-cells; class switching	AITD/GD (functional candidate region)
*TNFSF4* (OX40L)	Costimulatory ligand; T-cell survival	Reported autoimmune associations
*PADI4*	Citrullination enzyme (stronger RA signal)—occasional cross-disease hits	Reported in autoimmune cross-analyses
*ESRRA*	Nuclear receptor TF implicated by enrichment analysis in T1D	Identified by integrative analyses
*GSDMB*	Gasdermin family (inflammation/cell death)—locus sometimes implicated in autoimmune scans	Reported in multi-disease analyses
*SIRPG*	Receptor modulating T-cell–APC interactions	Reported in GWAS of autoimmune traits
*ZPBP2/ORMDL3*	Immune regulatory region; multiple autoimmune associations	Cross-disease signals
*CTLA4-CD28*	Modulates CTLA4/CD28 expression and T-cell activation	AITD
*GPR183* (EBI2)	B/T-cell positioning in lymphoid tissue	Reported in autoimmunity scans
*SLC22A4/5*	Reported in older linkage/GWAS work for autoimmunity	Various autoimmune associations (population dependent)
*ZFP36L1*	RNA-binding protein regulating cytokine mRNA stability	Implicated in immune regulation and reported in some analyses
*C1QTNF6*	Complement-related/immune signaling candidate	Reported in thyroid autoimmunity transcriptome/GWAS overlaps
*HLA-DR/DQ*, etc.	MHC antigen presentation	Autoimmune hypophysitis (primary)
*AIRE*	Central immune tolerance (thymic expression of self-antigens)	Gonadal autoimmune failure (ovary/testis) in APS1
*CYP21A2/CYP17A1*	Steroid biosynthesis in adrenal/ovary/testis	Autoimmune oophoritis/gonadal autoimmunity
*CTLA4/CD28*	T-cell costimulation/negative regulation	Hypophysitis (incl. immune-checkpoint-inhibitor induced)
*FOXP3*	Regulatory T-cell master regulator	Gonadal autoimmunity (conceptually)

**Table 2:** Reported genes and epigenetic mechanisms linked to autoimmune thyroid disease.

Gene/Locus	Physiological Function	Genetic Mechanisms/Variants	Epigenetic/Regulatory Aspects	Associated Phenotype/Subtype	References
*HLA-DR3*	presenting antigens to T-cells	HLA-DR (e.g., DRB1*03) shows the strongest and most reproducible association with GD; HLA signals for HT exist but are less uniform across cohorts	HLA-DRβ-Arg74 increases binding of modified TG peptides (e.g., under excess iodine).	GD (strong), HT (reported but less consistent), GD with ophthalmopathy; HLA patterns also relate to treatment response.	[2,6,10,14,15,17]
*PTPN22*	negatively regulates T-cell and B-cell signaling	Missense R620W (rs2476601; 1858C>T) is a replicated non-HLA risk allele that perturbs lymphocyte signaling thresholds and confers autoimmune risk	Promoter methylation changes in PTPN22 linked to HT have been reported.	GD and HT (ancestry-dependent: stronger in Europeans; variable in some Asian groups).	[1,10,14,20]
*CD40*	activating immune cells like B-cells and macrophages	Promoter variants (e.g., rs1883832 and linked 5′UTR polymorphisms) increase CD40 expression	Affects B-cell activation and antigen presentation.	Primarily GD: HT associations are inconsistent across studies.	[1,10,14]
*FOXP3*	development and function of regulatory T-cells	Promoter/intron SNPs (e.g., rs3761548/rs3761549) reported to alter FOXP3 expression/function in some cohorts	Variants and altered DNA methylation/acetylation at FOXP3 regulatory elements impair Treg stability and suppressive function.	GD, HT, and correlations with antibody titers/clinical course reported in some studies.	[1,13,15]
*CTLA4*	immune checkpoint for T-cells	Common SNPs (e.g., +49 A/G rs231775 and nearby markers) associate repeatedly with GD and broader AITD and are thought to impair CTLA-4 inhibitory function on T-cells	Alters T-cell costimulation.	GD, HT, AITD overall; linked to antibody levels and relapse risk in some cohorts.	[1,10,14]
*TG*	precursor for thyroid hormones (T4 and T3)	Multiple coding and non-coding SNPs and haplotypes (exons 10–12 cluster, exon 33, intron/intragenic SNPs) associate with AITD and TgAb positivity	Promoter SNP (−1623, rs180195) modifies an IRF-1 binding site and, together with IFNα, produces enhancer marks (H3K4me1) that up-regulate TG expression.	GD and HT; TgAb positivity and possible interaction with HLA-DRβ1-Arg74 to markedly increase risk.	[1,10,14,15,17]
*TPO*	catalyzes key steps in the production of thyroid hormones (T3 and *T*4)	SNPs (e.g., rs2071400, rs2071403, rs732609) have been associated with TPOAb levels, HT risk, and disease course	Limited data (promoter methylation, histone modifications).	HT and TPOAb positivity; influences on disease course reported.	[1,10,14]
*TSHR*	receptor on thyroid follicular cells that binds to thyroid-stimulating hormone (TSH)	Intronic-1 SNP cluster (e.g., rs179247, rs12101255/rs12101261) reproducibly associates with GD in multiple cohorts	Hypermethylation and altered histone marks reduce thymic expression; splicing changes generate immunogenic soluble isoforms.	GD (strong); some intron-1 alleles also linked to ophthalmopathy risk in specific studies.	[1,10,14,15,16,20]
Epigenetic mechanism	-	-	Global leukocyte hypomethylation (GD); H3K4me1 at TSHR/TG regulatory sites (IFNα-driven); altered histone demethylases (JMJD3: HT; LSD1: GO) in GO studies.	Distinct epigenetic signatures in GD vs. HT; potential prognostic utility. Thyroid hormone levels, treatment, and cell composition are important confounders to control for in methylation studies.	[9,13,16,18,20,21,22]

HLA: Human Leukocyte Antigen; GD: Grave’s Disease; HT: Hashimoto’s Thyroiditis; TG: Thyroglobulin; SNP: Single Nucleotide Polymorphism; AITD: Autoimmune Thyroid Disease; TgAb: Thyroglobulin Antibodies; TPOAb: Peroxidase Antibodies; IRF-1: Interferon Regulatory Factor-1; GO: Graves Oftalmopathy.

**Table 3:** Currently reported genetic associations in autoimmune hypophysitis.

Subtype/Entity	HLA Associations	Other Genetic/Molecular Findings	References
Lymphocytic hypophysitis	HLA-DR4, DR5, DQ8, DR53; celiac haplotypes (DQ8)	Pituitary autoantigens (*GH1/GH2*, *ENO1*, *PGSF1a/PGSF2*, *SCG2*, lactotroph autoantibodies)	[9,25,26]
ICI-induced hypophysitis	CTLA4–related: HLA-Cw12, HLA-DR15. PDCD1–related: HLA-DQB1*06:01*, *HLA-DPB1*09:01, HLA-DRB5*01:02. Others: HLA-DQ7, HLA-DPw9	Immune checkpoint gene polymorphisms (CTLA4, PDCD1). CNVs/VARs reported in TERT, SMAD3, JAK2, PRDM1, FAN1, CD274, UNG	[26]
Anti-PIT-1 hypophysitis	HLA class I presentation of POU1F1 epitopes implicated	Genetic regulation of B-cell autoantibody production; tumor-associated epigenetic dysregulation in thymoma	[2,9]

**Table 4:** Reported genetic and epigenetic mechanisms in parathyroid autoimmune disease.

Gene/Locus	Genetic Mechanisms/Variants	Epigenetic/Regulatory Aspects	Associated Phenotype/Subtype	References
*AIRE*	Classical recessive APS-1: >120 mutations; founder variants (p.R257X, p.R139X, p.Y85C, p.R203X, 13 bp exon 8 deletion).	Chromatin-dependent function via H3K4me0 binding; penetrance modified by sex, microbiota, and intrafamilial variability.	APS-1 (APECED) with hypoparathyroidism.	[4,4,5,27,33,34,35]
	Dominant non-classical APS-1: heterozygous PHD1 missense variants → milder, later-onset, incomplete penetrance.		Non-classical APS-1 with variable hypoparathyroidism.	[36,37]
*HLA*	Class I: HLA-A26:01; Class II: HLA-DRB101, HLA-DRB1*09.	-	Idiopathic/non-APS autoimmune hypoparathyroidism.	[27,30]
Other regulators	miR-220b represses AIRE (post-transcriptional).	microRNA and chromatin-based tolerance pathways.	Modifies both APS-1 and non-APS forms.	[37]
	FEZF2 enables AIRE-independent TSA expression.			

**Table 5:** Reported genetic and epigenetic associations in autoimmune adrenal disease.

Mechanism/Pathway	Genes/Loci	Diseases	Associations/Implications	References
Antigen presentation	*HLA-DR3–DQ2, HLA-DR4–DQ8*	AAD, APS-2	Strongest associations; linked to autoreactive T-cell activation. Epigenetic: global CD4^+^ hypomethylation.	[7,8,37,40]
Immune regulation/checkpoints	*CTLA4, PTPN22, CIITA, CLEC16A, CD274 (PD-L1), NLRP1, MICA/MICB*	AAD, APS-2	Associated with dysregulated T-cell signaling and immune checkpoint pathways.	[7,8]
Transcriptional/signaling regulators	*BACH2, SH2B3, UBASH3A, LPP, SIGLEC5*	AAD	GWAS associations; influence lymphocyte development and activation.	[7,8]
Central tolerance	*AIRE* (biallelic founder variants, hypomorphic/dominant-negative alleles)	APS-1, AAD (polygenic contribution), APS-2	Causal in APS-1; common variants associated with broader autoimmunity.	[30,33,35,36,37]
Rare/syndromic variants	*RAG1, TNFAIP3 (A20), LAT, IKZF2 (HELIOS)*	Adrenal autoimmunity	Reported in case series; suggest contribution to immune dysregulation.	[8]
Copy-number variation (CNV)	*UGT2B28* (low copy number more frequent in AAD); *ADAM3A* (high copy number associated with AAD)	AAD	UGT2B28: reduced steroid inactivation; ADAM3A: enhanced immune signaling	[3]

**Table 6:** Reported genetic and epigenetic mechanisms involved in autoimmune gonad disease.

Entity	Genetic Mechanism	Epigenetic/Regulatory Aspects	References
Autoimmune orchitis	AIRE mutations: impaired thymic expression of testis antigens; HLA alleles.	AIRE requires H3K4me0 binding for testis antigen display; failure → autoreactive T-cell escape.	[3,7,41,42,43]
Autoimmune oophoritis	*AIRE* mutations: loss-of-function variants are linked to ovarian autoimmunity; X chromosome CNVs (meiosis/DNA repair genes); *HLA-DR3* haplotype: associated with increased risk of POF; *FOXP3*: reduced FOXP3^+^ Tregs and Th17 skewing in autoimmune POI.	miR-21–Peli1 ↓ → reduced ovarian reserve, ↑ autoantibody burden miR-21–MSX1–Notch axis → ↓ granulosa apoptosis, ↑ steroidogenesis NF-κB–NLRP3 activation → granulosa pyroptosis; mesenchymal exosomes suppress pathway, improving ovarian function.	[40,41,44,45,46,47,48,49,50,51,52,53,54,55]

## Data Availability

All data are available upon request to the corresponding author.

## Abbreviations

The following abbreviations are used in this manuscript:

AITD	Autoimmune thyroid diseases
GD	Graves’ disease
HT	Hashimoto’s thyroiditis
APS-1	Autoimmune polyendocrine syndrome type 1
APECED	Autoimmune polyendocrinopathy-candidiasis-ectodermal dystrophy
TRAb	TSH-receptor autoantibodies
DZ	Dizygotic
MZ	Monozygotic
GO	Graves ophthalmopathy
ACTH	Corticotroph
TSH	Thyrotroph
LAH	Lymphocytic adenohypophysitis
LINH	Lymphocytic infundibulo-neurohypophysitis
LPH	Lymphocytic panhypophysitis
HLA	Human leukocyte antigen
ICI	Immune checkpoint inhibitors
*IIH*	*ICI-induced hypophysitis*
mAbs	Monoclonal antibodies
CTLA-4	Cytotoxic T-lymphocyte antigen 4
PD-1	Programmed cell death-1
CNVs	Copy number variations
VARs	Small variations
GH	Growth hormone
PRL	Prolactin
CaSR	Calcium-sensing receptor
PTH	Parathyroid hormone
CMC	Chronic mucocutaneous candidiasis
ASAs	Anti-sperm antibodies
TGM4	Transglutaminase-4
SVS2	Seminal vesicle secretory protein-2
POI	Primary ovarian insufficiency
